# Assessment of parenteral estradiol and dihydroxyprogesterone use among other feminizing regimens for transgender women: insights on satisfaction with breast development from community-based healthcare services

**DOI:** 10.1080/07853890.2024.2406458

**Published:** 2024-09-20

**Authors:** Camila Toffoli Ribeiro, Ísis Gois, Mariana da Rosa Borges, Lucas Garcia Alves Ferreira, Matheus Brandão Vasco, João Guimarães Ferreira, Taciana Carla Maia, Magnus Régios Dias-da-Silva

**Affiliations:** a Hospital de Clínicas da Universidade Federal de Uberlândia, Uberlândia, Brazil; b Centro de Referência e Assistência Integral para a Saúde Transespecífica (CRAIST) HC/UFU, Uberlândia, Brazil; c Núcleo TransUnifesp (NTU), Universidade Federal de São Paulo, São Paulo, Brazil; d Laboratory of Molecular and Translational Endocrinology, Department of Medicine, Escola Paulista de Medicina, Universidade Federal de São Paulo, São Paulo, Brazil

**Keywords:** Transgender women, gender-affirming hormone therapy, estradiol enanthate, dihydroxyprogesterone, transgender primary care, community-based healthcare, satisfaction with breast development, travestis

## Abstract

The practice of hormone therapy is crucial in aligning secondary sex characteristics with the gender identity of transgender adults. This study examines the effects of a commonly used injectable hormone combination, specifically estradiol enanthate with dihydroxyprogesterone acetophenide (EEn/DHPA), on serum hormonal levels and self-reported satisfaction with breast development in transwomen. Our research focused on a retrospective longitudinal study involving a large cohort of transwomen evaluated between 2020 and 2022, comprising 101 participants. We assessed serum levels of estradiol (E2), testosterone (T), luteinizing hormone (LH), and follicle-stimulating hormone (FSH), comparing the EEn/DHPA hormonal regimen with other combined estrogen-progestogen (CEP) therapies. Additionally, a subset of 43 transwomen completed a 5-question survey to evaluate self-reported satisfaction with breast development using Tanner scales. Our findings indicated that participants using the EEn/DHPA regimen exhibited significantly higher serum E2 levels (mean: 186 pg/mL ± 32 pg/mL) than those using other therapies (62 ± 7 pg/mL), along with lower FSH levels, but no significant differences in T and LH levels. Concerning satisfaction with breast development, 76% reported increased fulfillment with breast augmentation while using EEn/DHPA. These results suggest that an injectable, low-cost EEn/DHPA administered every three weeks could serve as an alternative feminizing regimen, particularly considering the extensive long-term experience of the local transgender community. Further longitudinal studies on the efficacy of feminizing-body effects and endovascular risks of various parenteral CEP types are warranted to improve primary healthcare provision for transgender persons.

## Introduction

Transgender individuals experience a mismatch between their gender identity and the sex assigned at birth, forming a diverse demographic that presents challenges in providing comprehensive healthcare [1–4]. Cultural competence among health professionals engaging with the transgender community must encompass specific attitudes to empower transgender individuals in decision-making processes [5,6]. This is particularly crucial when considering diverse gender-affirming hormone therapy (GAHT) regimens and surgical interventions [7,8]. Decision-making regarding these therapies significantly varies across multi-professional healthcare services.

GAHT serves as a fundamental element in the endocrine care continuum for transgender individuals seeking bodily alterations to align with their gender identity. Like every intervention for transgender people, GAHT must be flexible and individualized since there is a spectrum of gender identities, including non-binary gender identity [9]. For transwomen, a typical regimen often involves a combination of estrogens and antiandrogens. Their objectives commonly include eliminating body hair growth, fostering breast formation and augmentation, and achieving a more feminine body fat distribution. A near-complete reduction of androgenic bodily characteristics through estrogen intake is frequently pursued to realize these modifications. Estrogens can suppress gonadotropin output and consequently curtail endogenous testosterone production. However, combined therapy can yield more effective outcomes, employing one hormone to suppress testosterone secretion or action alongside another, inducing feminizing changes such as estrogen [2,10].

Various antiandrogens function to either inhibit testosterone biosynthesis/action or reduce endogenous testosterone levels by impeding the hypothalamic-pituitary-gonadal axis or both. The most used ones are spironolactone (SPL) and cyproterone acetate (CPA) [11].

The addition of progestogens in the regimen for transwomen may positively impact breast augmentation and body feminization [12]. However, this effect remains speculative and needs further research [13]. Our group has replicated the use of estradiol plus progestin with antiandrogen effects in rodent models, revealing no adverse effects but rather significant responses in feminizing body changes [14].

Injectable combined estrogens with progestogens (CEP) have long been widely used in Brazil and other Latin American countries, predominantly among ciswomen as an injectable contraceptive and by Brazilian transgender women and *travestis* as GAHT [8]. Despite the absence of recognition by the Endocrine Society as an alternative hormonal regimen due to concerns regarding thrombogenicity and challenges in routine monitoring through blood testing, the prevalent use of CEP necessitates evaluating its regimen recommendations. This has led our research to delve deeper into understanding CEP regimens, considering the experiences of *travestis* amidst distinct sociocultural lifestyles and limited access to public endocrinological care services [15,16]. Hence, our objective is to elucidate our observations in monitoring trans individuals utilizing CEP regimens by evaluating hormone levels and self-perceived satisfaction in breast development within a cohort of transwomen employing the most common injectable CEP, namely estradiol enanthate with dihydroxyprogesterone acetophenide (EEn/DHPA) and comparing these observations with other GAHT regimens.

## Subjects and methods

### Characterization of cohort

We conducted a retrospective longitudinal study of a cohort of Brazilian transfeminine participants, a community self-referred as transwoman and *travestis* [15], between 2020 and 2023 at the Transgender Outpatient Clinics of Universidade Federal de Uberlândia (UFU) and Universidade Federal de São Paulo (UNIFESP). Among the initial participants, 92 received follow-up at UNIFESP, while 54 attended UFU Transgender Clinics. The study adhered to the ethical regulations of the university’s research protocols and specific guidelines outlined by Brazil’s public healthcare system, the Unified Health System (SUS).

### Comparison of hormonal regimens

For the comparative analysis of hormonal regimens, 29 individuals were excluded due to conflicting biochemical or inconsistent clinical information. This exclusion applied to those who lacked baseline hormone level measurements or had irregular follow-ups in the last two years. Additionally, some reported mixed hormonal regimens or changed regimens within one year, lacking confirmatory lab data in medical records. Those hospitalized for severe morbidities were also excluded. One hundred and one out of 146 participants remained in the study for at least 24 months, providing serum hormonal data (estradiol, testosterone, LH, FSH) during follow-ups while utilizing various GAHT regimens. All enrolled trans women were either on GAHT or expressed a desire to undergo therapy and willingly participated in the study.

Regarding the comparison of Combined Estradiol/Progestogen (CEP) regimens, we divided the cohort into two groups: Group 1 (*N* = 53), comprising individuals using EEn/DHPA, and Group 2 (*N* = 48) involving other CEP formulations. The latter included oral, transdermal, or injectable CEP hormonal combinations, such as 4 mg estradiol valerate and 100 mg cyproterone acetate taken orally daily (*N* = 25); 3 mg gel-pump transdermal estradiol with 100 mg CPA taken daily (*N* = 9); 50–100 µg transdermal patch estradiol applied twice a week with 100 mg SPL (*N* = 5); 2 mg estradiol valerate plus 1 mg CPA taken daily (*N* = 3); monthly injectable 5 mg estradiol valerate plus 50 mg norethisterone acetate NETA (*N* = 2); and oral 1 mg 17ß-estradiol plus 0.5 mg NETA (*N* = 2).

### Satisfaction with breast development

**Figure 1.**
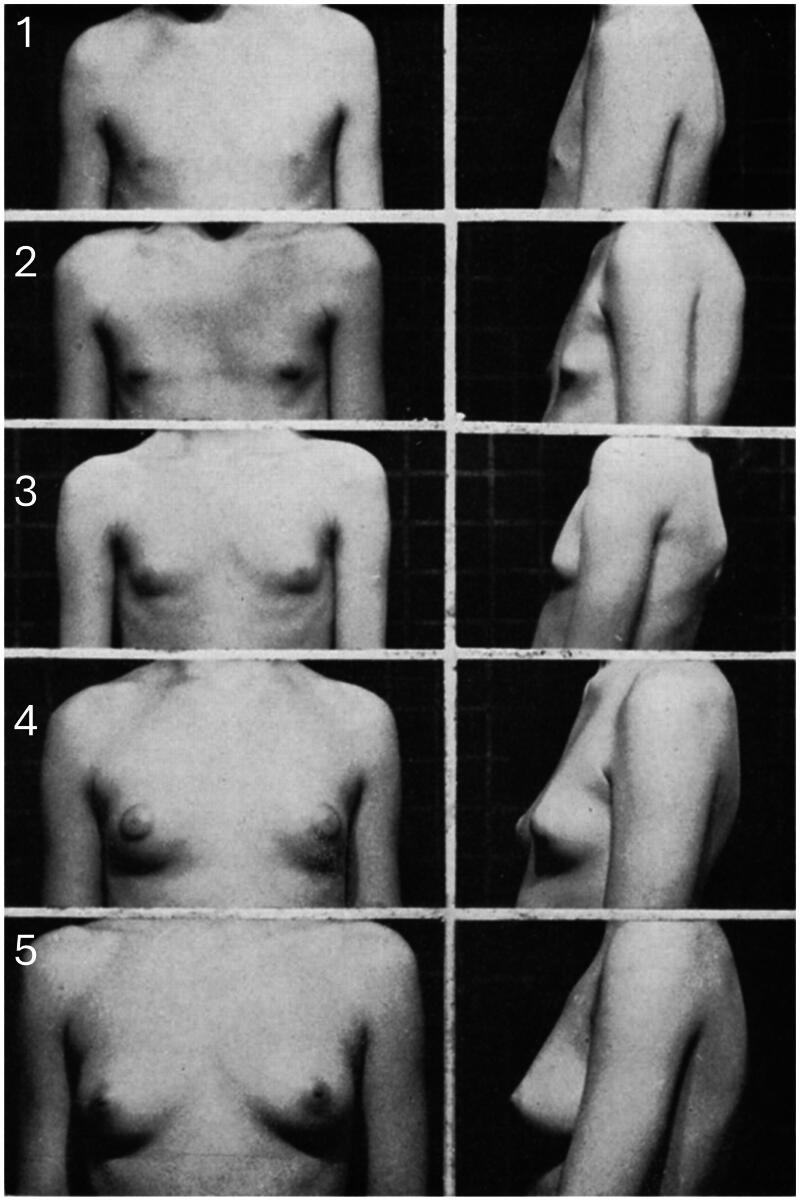
Representation of breast growth Tanner stages. The panel was utilized to standardize the responses from transwomen undergoing gender-affirming hormone therapy (GAHT).

The phase involving self-reported satisfaction with breast development included the enrolment of 45 transwomen to respond to a 5-question online survey. Two participants who had reported the use of ethinylestradiol were excluded. The questions covered their interest in documenting their perception of breast development before and after hormone use based on Tanner scale representations (Figure 1), previous experiences with injectable hormones containing estradiol plus progestin (e.g. PERLUTAN^®^, DAIVA^®^, and UNOCICLO^®^), and which regimen would have worked better for breast augmentation.

### Serum hormonal measures

Participants routinely provided a 5-mL blood sample during outpatient clinic visits at university reference centers (UFU and UNIFESP). E2 (pg/mL) and Testosterone (ng/dL) were measured using a competitive electrochemiluminescence immunoassay (Roche Cobas^®^). LH (IU/L) and FSH (IU/L) serum levels were determined through electrochemical luminometry (Roche Cobas^®^).

### Estradiol enanthate pharmacokinetics curve

Utilizing a previously published meta-analysis method of estradiol concentration-time data from publicly available information on cisgender women who had used EEn or EEn/DHPA [17], we reanalyzed and integrated data from various studies. A unified single-dose curve for 30 days was created. We employed least squares regression for studies with four or more concentration-time data points (solid lines). We manually adjusted other studies with three data points to fit into a single-dose curve.

Each study’s data were adjusted for baseline estradiol levels or endogenous estradiol production and then normalized by 10 mg. The V3C Fitter and Desmos tools, accessible online at https://alyw234237.github.io/injectable-e2-simulator/v3c-fitter/ and https://www.desmos.com/calculator/ndgvp2avhj?lang=pt-BR respectively, were utilized for fitting the three-compartment pharmacokinetic model. Estradiol levels from transgender women on EEn/DHPA in this study were presented using a box plot graph featuring percentiles at 10, 25, 50, 75, and 90.

### Statistical analysis

The results were presented as mean ± standard error of the mean and analyzed using the Mann-Whitney test because our data did not meet the criteria for normality and homogeneity (Prism 6.0, GraphPad). We utilized the Spearman correlation test to evaluate linear regression. Breast development Tanner stages were illustrated through individual values and means, with assessments performed using the Wilcoxon test (paired test). Statistically significant differences were determined at *p* ≤ 0.05.

## Results

We collected clinical and laboratory data from 101 out of 146 invited transfeminine people undergoing hormone treatment at the Transgender Outpatient Clinics of UFU and UNIFESP (Table 1). Additionally, 43 participants voluntarily responded to the breast satisfaction survey (Table 2). The study participants had a mean age of 35.8 ± 9.98 and had undergone hormone therapy for an average of 8.9 ± 6.9 years. Among participants using parenteral EEn/DHPA, the mean application frequency was 24.6 ± 6.6 days, with a predominant mode of applications every 30 days.

**Table 1.** Summary of hypothalamic-pituitary-gonadal axis profile of trans persons retrospectively followed in this study using feminizing hormone regimens.

Measurement	Hormone regimen type				*Mann-Whitney*	
	EEn/DHPA		Others			
	*N*	Mean ± SEM	*N*	Mean ± SEM	*U*	*p*
LH (UI/L)	50	1.3 ± 0.3	40	2.9 ± 0.8	810.5	0.1211
FSH (UI/L)	48	1.5 ± 0.3	39	5.2 ± 1,8	698	0.0415
Testosterone (ng/dL)	52	62.9 ± 20.1	42	94.9 ± 24.7	927	0.2112
Estradiol (pg/mL)	52	186.4 ± 32.8	47	62.2 ± 6.9	636	<0.0001

Detailed data are listed in Supplemental Table 1.

**Table 2.** Self-reported satisfaction with breast development using the Tanner stage scale.

Variables	Frequency (n)	Percentage (%)
Influence of hormone use on breast development		
No effect	0	0.0
Little improvement	10	23.3
Regular improvement	22	51.2
Excellent improvement	10	23.3
Do not know	1	2.2
Tanner breast stage before the use of hormone (GAHT)		
1	22	51.2
2	8	18.6
3	6	13.9
4	3	7.0
5	4	9.3
Tanner breast stage after the use of hormone (GAHT)		
1	2	4.8
2	0	0.0
3	14	32.6
4	14	32.6
5	13	30.2
Use or passed used of EEn/DHPA		
Yes	37	86.0
No	6	14.0
GAHT regimen with the most self-perceived breast development		
EEn/DHPA	28	65.1
Other hormones	9	20.9
Do not know	6	14.0
General satisfaction with breast development		
Dissatisfied	9	20.9
Partially satisfied	17	39.6
Satisfied	10	23.3
Very satisfied	7	16.3
**TOTAL**	**43**	**100**

Forty-three transwomen responded to a 5-question online survey whose questions covered the interest in documenting their perception of breast development before and after hormone use based on Tanner scale representations (Figure 1). Participants documented previous experiences with hormones containing estradiol plus progestin and which regimen would have worked better for breast augmentation.

### Hormonal levels during the follow-up of feminizing regimens

**Figure 2.**
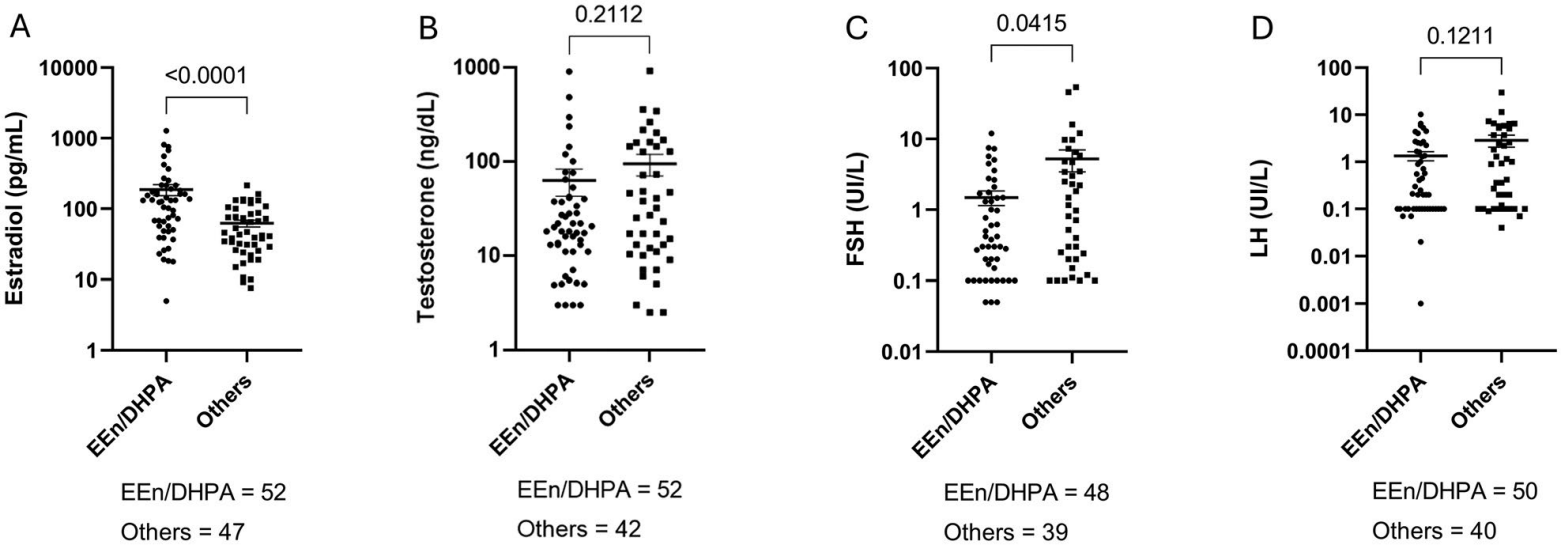
Hormonal levels during the follow-up of observed feminizing regimens. Levels of estradiol in pg/mL (a), total testosterone in ng/dL (B), FSH in UI/L (C), and LH in UI/L (D) among transwomen taking EEn/DHPA compared to other hormonal feminizing regimens (others). Bars represent the median.

Scatter plot graphs depicted the measurement of sex hormones (Figure 2). Serum estradiol levels in the EEn/DHPA group (mean: 186.4 pg/mL ± 32.8 pg/mL) were significantly higher than those in the group using other therapies (62.2 ± 6.9 pg/mL) (Figure 2(A)). Within the EEn/DHPA group, serum FSH levels were significantly lower compared to the other group (Others) (Figure 2(B)). However, no significant difference was found between the groups concerning testosterone (Figure 2(C)) and LH (Figure 2(D)) levels.

### Self-reported evaluation of satisfaction with breast development

**Figure 3.**
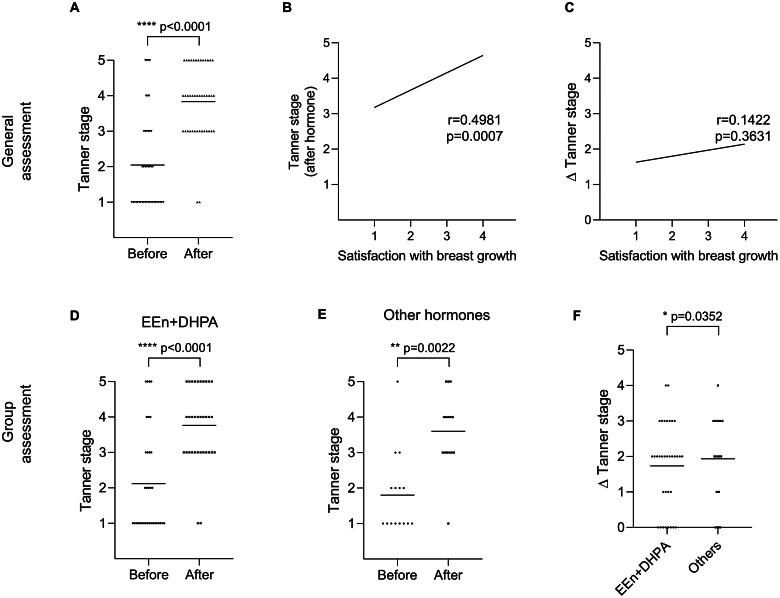
Self-reported satisfaction with breast development among transwomen undergoing hormonal feminizing regimens. (A) Comparison of self-reported Tanner stage before and after hormone therapy using the Wilcoxon (paired) test. Data is presented as individual values and means. (B) Exploration of the relationship between post-hormone Tanner stage and satisfaction level using the Spearman correlation test with linear regression. (C) Analysis of the correlation between satisfaction level and variance (Δ) in self-reported Tanner stage using the Spearman correlation test with linear regression. Satisfaction levels range from 1 – no effect; 2 – Slight improvement; 3 – improvement, but regression after discontinuation; and 4 – significant improvement. Self-reported assessment of breast Tanner stage before and after using EEn/DHPA (D) or other hormones (E), and the comparison of the variance (Δ) of the self-reported Tanner stage between EEn/DHPA and other groups (F) by applying the Wilcoxon test. The general assessment of satisfaction with breast development is presented in panels (a), (B), and (C) with 43 participants. The group-specific assessments comprise EEn/DHPA (*n* = 28) and other hormone users (*n* = 9), depicted in panels (D), (E), and (F). To ensure statistical accuracy, participants who responded, ‘don’t know’ (*n* = 6) were evenly distributed between the two groups.

In the general analysis encompassing all participants undergoing either EEn/DHPA or alternative hormonal regimens, self-reported satisfaction with breast development notably improved following the hormonal feminizing regimen (Figure 3(A)). Notably, a positive correlation emerged between the post-hormone usage Tanner stage and the degree of satisfaction with breast augmentation (Figure 3(B)). Conversely, no significant correlation was discerned between Tanner stage variance (Δ) alterations and breast growth satisfaction (Figure 3(C)). The self-reported evaluations of the breast Tanner stage before and after employing EEn/DHPA (Figure 3(D)) or other hormonal agents (Figure 3(E)) indicated comparable outcomes. Similarly, comparing the variance in the self-reported Tanner stage between the EEn/DHPA and Other groups (Figure 3(F)) yielded nonsignificant differences when assessed through the Wilcoxon test.

**Figure 4.**
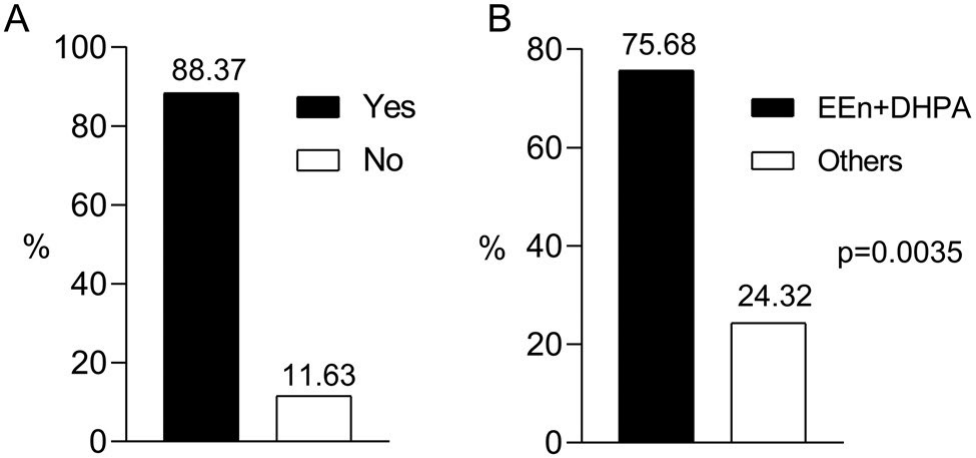
Assessment of EEn/DHPA parenteral use compared to other hormonal regimens concerning self-reported satisfaction with breast development. (A) Evaluation of self-reported satisfaction with breast development among transgender women who have used EEn/DHPA within the last 2 years. (B) Comparison of the proportions of participants reporting better breast development under EEn/DHPA use or other regimens against an even distribution (50% and 50%) using a binomial test. Considering previous usage patterns and statistical analysis, this figure provides insight into the comparative effectiveness of EEn/DHPA parenteral use versus other hormonal regimens regarding self-reported satisfaction with breast development. EEn/DHPA (*n* = 28); Others (*n* = 9); not known (*n* = 6).

However, a substantial majority of participants (88.37%) reported prior utilization of EEn/DHPA during the hormonal feminizing regimen (Figure 4(A)), with 75.68% expressing superior breast development while under EEn/DHPA compared to alternative hormone combinations (Figure 4(B)).

### Pharmacokinetics of injectable estradiol enanthate

**Figure 5.**
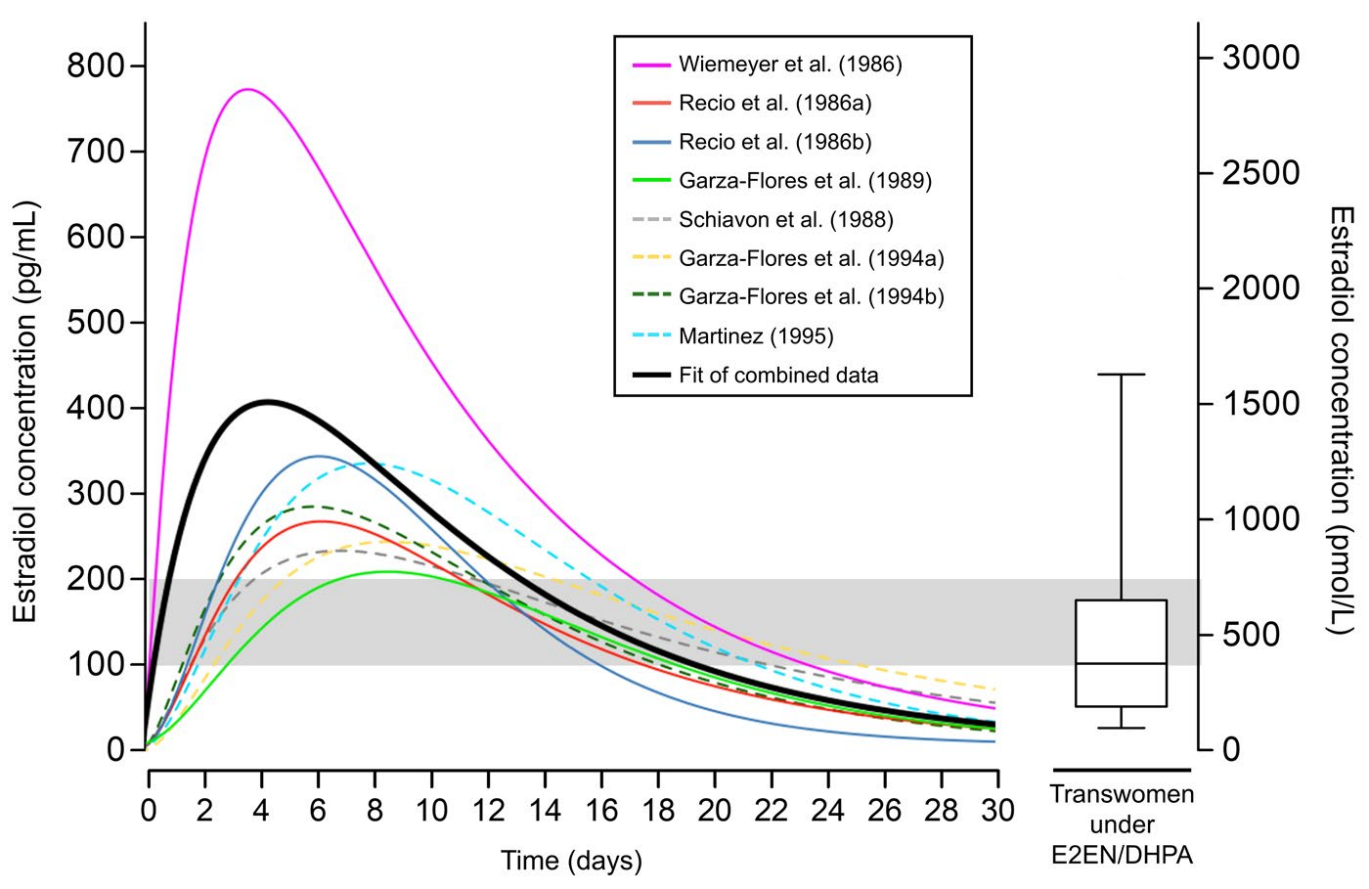
Meta-analysis of estradiol concentration-time data from cisgender women under EEn alone or EEn/DHPA. Fitted data curves from various studies individually and combined into a single-dose curve over 30 days were generated based on an informal meta-analysis of published estradiol concentration-time data from cisgender women under EEn or EEn/DHPA [17]. For studies with four or more concentration-time data points (solid lines) and the fit of combined data (thick black line), least squares regression to a three-compartment pharmacokinetic model was employed. A single-dose curve was manually adjusted for studies with three data points (dashed lines). Data from each study were adjusted for endogenous estradiol production via baseline or trough estradiol levels subtraction and normalized by 10 mg. The graph illustrates estradiol levels from the transwoman cohort in a boxplot. The shaded area represents the optimal target range for estradiol levels in transwomen under hormone therapy. The boxplot graph displays the percentiles 10, 25, 50, 75, and 90 for estradiol levels of transwomen under EEn/DHPA in this study (*N* = 53).

Serum estradiol levels in trans women using EEn/DHPA reached the target levels for this population during hormone therapy, a trend not observed in participants using other feminizing hormone therapies (Table 1). The boxplot graph (Figure 5) illustrates that the median estradiol levels in trans women using EEn/DHPA fell within this population’s expected average range values (100–200 pg/mL).

## Discussion

The cornerstone of gender-affirming feminizing therapy for transfeminine individuals lies in the administration of estrogen and anti-androgen. This therapeutic approach encompasses a spectrum of hormone doses and types, with considerations spanning availability, cost, efficacy, and safety. However, at the heart of this decision-making process remains a crucial acknowledgment of the transitioning individual’s self-perception and their journey toward gender affirmation.

Our study represents a pioneering contribution to the literature by demonstrating that Brazilian trans women undergoing EEn/DHPA therapy achieved estradiol levels comparable to those observed during the follicular phase in cisgender women. Moreover, these individuals expressed heightened satisfaction with breast development while utilizing EEn/DHPA (71.68%). This discovery holds significant implications for tailoring hormone prescription regimens within primary healthcare settings.

Our study further noted that DHPA demonstrates comparable efficacy to cyproterone or other anti-androgens in achieving optimal LH pituitary suppression and reducing testosterone levels. EEn/DHPA, an affordable injectable contraceptive widely accessible in South American countries, presents a promising avenue for attaining target hormone levels among transfeminine individuals.

Additionally, our investigation, which reviewed pharmacokinetic data, supports the potential implementation of EEn/DHPA in a 21-day regimen to sustain optimal estradiol levels. While alternative medications exist to inhibit testosterone production and action, their availability varies based on regional healthcare provider systems. Commonly prescribed options such as CPA and SPL, as well as alternatives like finasteride, aim to address testosterone-related concerns. Although injectable long-acting gonadotropin-releasing hormone agonists offer effectiveness and convenience, their widespread utilization encounters challenge due to cost considerations and regulatory approval constraints.

The development of female body traits in transgender women primarily relies on estrogen, ideally transdermal 17ß or micronized estradiol, though its dispensation by public healthcare providers varies [2,18]. Oral and transdermal 17ß-oestradiol are extensively prescribed worldwide [19,20], yet injectable forms, when available, offer convenience and potentially higher estrogen levels by bypassing the first hepatic metabolism *via* lymphatic system drainage. EEn/DHPA, commonly used as a long-lasting injectable contraceptive [21–23], has found application in feminizing hormone therapy for transfeminine people, notably in *travestis* in Brazil [7,8,24,25]. The inclusion of progestin in feminizing hormone therapy remains a subject of debate [26]. While some advocate for its positive impact on areolar and body shape development, others contest its role, raising concerns about associated risks, especially with newer progestins [27,28].

A significant portion of our understanding regarding the effects of progestogens stems from research conducted in cisgender populations. In menopausal cis women, progesterone is utilized to mitigate the risk of endometrial hyperplasia and malignant transformation during estrogen therapy [29]. Combined estrogen-progestogen (CEP) regimens, essential for women with an intact uterus, elevate the risk of thromboembolism (TE) when compared to estrogen alone, except for micronized progesterone, which appears to have a neutral impact [30]. Moreover, the method of estrogen delivery is pivotal, as there is no TE risk associated with isolated transdermal 17ß-estradiol treatment [31]. The type of estrogen used also holds significant importance. In the realm of contraception, it is widely acknowledged that synthetic estrogens like ethinylestradiol (EE) pose a higher risk compared to estradiol valerate or 17β -estradiol [32].

The progestins in combined oral contraceptives (COC) modulate the extent to which estrogen stimulates the synthesis of liver coagulation factors. Androgenic progestins found in second-generation COC can counteract ethinylestradiol effects on the liver and can be women’s first choice in some countries for safety reasons [33]. Moreover, the risk of venous thromboembolism (VTE) in the general population is influenced by both drug-related factors, such as the duration of exposure and route of administration, and user-related factors, including smoking, age, obesity, and genetic predisposition to thrombophilia [34].

In the transgender population, the objectives of feminizing hormone therapy (FHT) regimens vary significantly despite limited literature [35] conducted an observational study involving 2509 trans women, revealing that the prescription of progestogens in gender-affirming hormone therapy (GAHT) was linked to a nearly threefold increase in the risk of thromboembolism, with medroxyprogesterone acetate appearing to pose a higher risk. However, the study’s sample size was insufficient to definitively support this conclusion. A recent systematic review on the use of progestins in GAHT, encompassing 10 articles, with 9 focusing on cardiovascular aspects, observed increased thromboembolic events and decreased levels of high-density lipoprotein (HDL). However, there was significant heterogeneity in the data, including variations in doses, routes of administration, and types of estrogens and progestins [13].

Furthermore, the dosages of progestogens in CEP regimens are considered relevant to reported side effects and risks. In a cohort of 882 trans women [36] demonstrated that even low doses of cyproterone, as low as 10 mg, achieved adequate hypothalamic suppression. Nevertheless, they reported cases of superficial venous thrombosis (SVT) and pulmonary thromboembolism (TE), particularly in participants with pre-existing clinical morbidities. In our retrospective cohort, we observed a case of thrombophlebitis in the leg of a heavy smoker using CEP, likely attributable to multiple industrial liquid silicone (ILS) implants in the gluteal region, resulting in sclerosing lipogranulomatosis.

Considering the impact of estrogen delivery route and types on TE risk, it is biologically plausible to assume that estrogen administered parenterally in a CEP regimen could result in an acceptable low VTE risk. Surprisingly, research on the risk of TE in transfeminine individuals on EEn/DHPA is scarce, with minimal reported cardiovascular events in cisgender women [21–24,37]. Progesterone plays a crucial role in breast development, with observations indicating its necessity for ductal branching and eventual maturation, leading to substantial differences in female breast characteristics [38]. Notably, the use of (micronized) progesterone in conjunction with estrogen aims not only to achieve satisfactory estradiol levels for promoting more rapid feminization and optimal breast maturation but also to replicate the antiandrogen progesterone’s effects by effectively suppressing LH and, thereby, better reducing gonadal testosterone synthesis [39].

We found that DHPA, the most used progestin in injectable CEP formulations, behaves similarly to other progestins [40–44], which may explain the higher satisfaction with breast development observed in the EEn/DHPA group. As research in transgender healthcare progresses, gaining a deeper understanding of the role of other antiandrogenic progestins prescribed for transgender individuals beyond oral cyproterone becomes increasingly necessary [45–47].

However, this study has limitations. Firstly, its retrospective design may have resulted in missing data on mild adverse effects and selection bias regarding the GAHT regimen most commonly chosen by the population and maintained by our physicians. Secondly, the comparative group encompassed those in use with heterogeneous therapeutic options, thus potentially interfering with general comparisons. Thirdly, many participants were excluded due to the use of mixed GAHT regimens, leading to fewer cases for statistical analysis. Therefore, our results should be reviewed in prospective long-lasting studies with a larger cohort using EEn/DHPA alone or featuring a comparative group comprising estrogen-cyproterone users.

In conclusion, our long-term cohort study suggests that administering parenteral estradiol enanthate with dihydroxyprogesterone acetophenide every three weeks could serve as a practical option for feminizing hormone regimens in transgender women. Nonetheless, adopting an individualized approach that takes into account each individual’s goals, response to prior hormone therapies, and medical history is crucial. This personalized approach is central to improving healthcare provision and ensuring optimal outcomes in bodily changes. By continuing to explore and refine hormone therapy regimens, we can better support the health and well-being of transgender individuals on their gender-affirming journey.

## Supplementary Material

Supplemental_Table_1.docx

## Data Availability

Data supporting the results or analyses presented are available upon reasonable request. Please contact the corresponding author to arrange access to study datasets.
