# Supplementary material for: Assessment of parenteral estradiol and dihydroxyprogesterone use among other feminizing regimens for transgender women: insights on satisfaction with breast development from community-based healthcare services
Source: Ann Med. 2024 Sep 20;56(1):2406458. doi: 10.1080/07853890.2024.2406458 (PMC11418065; doi:10.1080/07853890.2024.2406458)
Supplement: Supplemental_Table_1.docx [file IANN_A_2406458_SM6082.docx]

**Supplemental Table 1.**

| **Participant** | **GAHT** | **LH (UI/L)** | **FSH (UI/L)** | **Testosterone (ng/dL)** | **Estradiol (pg/mL)** |
| --- | --- | --- | --- | --- | --- |
| 1 | EEn/DHPA | 2.68 | 2.09 | 53.00 | 808 |
| 2 | EEn/DHPA | 0.9 | 3.6 | 16 | 132 |
| 3 | EEn/DHPA | 0,45 | 1,69 | 22 | 423 |
| 4 | EEn/DHPA | 0.1 | 0.1 | 22 | 94.5 |
| 5 | EEn/DHPA | 4.4 | 1.3 | 119 | 270 |
| 6 | EEn/DHPA | 0.02 | 0.1 | 28 | 133.8 |
| 7 | EEn/DHPA | 4.5 | 12 | 483.00 | 162 |
| 8 | EEn/DHPA | 0.2 | 0.3 | 27 | 1277 |
| 9 | EEn/DHPA | 5.9 | 7.5 | 237 | 5 |
| 10 | EEn/DHPA | 2.7 | 7.3 | 26 | 48.6 |
| 11 | EEn/DHPA | 1 | 0.3 | 37.55 |  |
| 12 | EEn/DHPA | 0.1 | 0.2 | 13 | 81 |
| 13 | EEn/DHPA | - | - | 5.1 | 17.9 |
| 14 | EEn/DHPA | 0.07 | 0.3 | 17.3 | 214.7 |
| 15 | EEn/DHPA | 10.2 | - | 902 | 39.1 |
| 16 | EEn/DHPA | 0.2 | 0.27 | 18 | 192 |
| 17 | EEn/DHPA | 6.51 | 5.68 | 5.47 | 50.4 |
| 18 | EEn/DHPA | 0.001 | 0.05 | 20 | 368 |
| 19 | EEn/DHPA | 0.2 | 0.2 | 17.4 | 755 |
| 20 | EEn/DHPA | 0.21 | 0.97 | 5 | 217 |
| 21 | EEn/DHPA | 0.3 | 0.3 | 11 | 675.4 |
| 22 | EEn/DHPA | 0.07 | 1.46 | 40 | 162 |
| 23 | EEn/DHPA | 0.1 | 0.1 | 3 | 56.68 |
| 24 | EEn/DHPA | 0.1 | 0.42 | 18 | 131.8 |
| 25 | EEn/DHPA | 0.1 | 0.1 | 13 | 181.1 |
| 26 | EEn/DHPA | 0.1 | 0.1 | 16 | 252.4 |
| 27 | EEn/DHPA | 1.3 | 0.77 | 77 | 215 |
| 28 | EEn/DHPA | - | 0.2 | 13.6 | 68 |
| 29 | EEn/DHPA | 0.57 | 0.17 | 11 | 154.2 |
| 30 | EEn/DHPA | 2.48 | 2.65 | 64.2 | 23.08 |
| 31 | EEn/DHPA | 4.07 | 2.76 | 143 | 38.86 |
| 32 | EEn/DHPA | 0.1 | 0.05 | - | 57 |
| 33 | EEn/DHPA | 2.26 | 1.76 | 6 | 47.8 |
| 34 | EEn/DHPA | 0.1 | 0.1 | 3 | 144.2 |
| 35 | EEn/DHPA | 0.41 | - | 11 | 72.28 |
| 36 | EEn/DHPA | 1.35 | 1 | 37 | 74.55 |
| 37 | EEn/DHPA | 0.1 | 0.38 | 3 | 105.4 |
| 38 | EEn/DHPA | 0.1 | 0.62 | 13 | 25.88 |
| 39 | EEn/DHPA | 1.66 | 1.31 | 76 | 67.79 |
| 40 | EEn/DHPA | 0.1 | 0.1 | 5 | 123.6 |
| 41 | EEn/DHPA | 0.1 | 0.42 | 18 | 131.8 |
| 42 | EEn/DHPA | 0.55 | 0.5 | 21.9 | 172 |
| 43 | EEn/DHPA | 0.1 | 0.1 | 3 | 101.8 |
| 44 | EEn/DHPA | 0.2 | 0.1 | 20.52 | 173.1 |
| 45 | EEn/DHPA | 0.1 | 0.05 | 42 | 126 |
| 46 | EEn/DHPA | - | - | 7 | 137 |
| 47 | EEn/DHPA | 1.62 | 4.49 | 33.6 | 27.19 |
| 48 | EEn/DHPA | 2.55 | 0.15 | 41 | 36.76 |
| 49 | EEn/DHPA | 0.1 | - | 14.6 | 19.09 |
| 50 | EEn/DHPA | 5.25 | 5.07 | 100.5 | 65.96 |
| 51 | EEn/DHPA | 0.25 | 0.6 | 4.89 | 18.31 |
| 52 | EEn/DHPA | 0.1 | 0.28 | 28.22 | 555.5 |
| 53 | EEn/DHPA | 0.71 | 1.48 | 296.1 | 160 |
| 54 | Other | 11.41 | 45.9 | 160.57 | 15 |
| 55 | Other | 0.9 | 0.4 | 5 | 41 |
| 56 | Other | 0.09 | - | 12 | 37.9 |
| 57 | Other | 7.25 | 16 | 262 | 26 |
| 58 | Other | 3.7 | 4.7 | 159 | 19 |
| 59 | Other | 2.2 | 1.48 | - | 135 |
| 60 | Other | 0.42 | 0.52 | 72 | 72 |
| 61 | Other | 5.86 | 0.71 | 358 | 10.7 |
| 62 | Other | 0.1 | 0.24 | - |  |
| 63 | Other | - | - | 26.76 | 29 |
| 64 | Other | 0.27 | 0.25 | 6 | 9.2 |
| 65 | Other | - | 9.7 | 145 | 31 |
| 66 | Other | - | - | 13 | 87.2 |
| 67 | Other | 0.12 | 0.3 | 2.5 | 76.6 |
| 68 | Other | - | 9.7 | 145 | 31 |
| 69 | Other | 3.7 | 2.3 | 202.6 | 69 |
| 70 | Other | 0.2 | 0.2 | - | 43 |
| 71 | Other | 5.25 | 4.8 | 127 | 38.18 |
| 72 | Other | 5.7 | 7.29 | 47.3 | 33.85 |
| 73 | Other | 1.83 | 3.48 | 128 | 7.61 |
| 74 | Other | 0.04 | 0.11 | 22.99 | 67 |
| 75 | Other | - | - | 3 | 64.84 |
| 76 | Other | 0.2 | 0.97 | 31.82 | 39 |
| 77 | Other | 6.54 | 6.52 | - | 73.8 |
| 78 | Other | - | - | 918 | 41.75 |
| 79 | Other | 0.1 | 0.15 | 15 | 25.46 |
| 80 | Other | 0.93 | 0.12 | 40.7 | 119.7 |
| 81 | Other | 1.01 | - | 11 | 16.94 |
| 82 | Other | 2.6 | 3 | 169.3 | 10 |
| 83 | Other | 0.1 | 0.1 | 7 | 100.2 |
| 84 | Other | 2.47 | 3.42 | - | 214 |
| 85 | Other | 0.1 | 0.1 | - | 161.9 |
| 86 | Other | 1.15 | 1.83 | 10 | 75.5 |
| 87 | Other | 29.66 | 53.88 | 25 | 105.1 |
| 88 | Other | 1.3 | 0.81 | 48.26 | 22 |
| 89 | Other | 0.1 | 0.1 | 16.97 | 47 |
| 90 | Other | 5.11 | 2.5 | 73 | 34.55 |
| 91 | Other | 6.4 | 5.82 | 343 | 32 |
| 92 | Other | - | - | 46 | 53 |
| 93 | Other | - | - | 38 | 84 |
| 94 | Other | 6.46 | 12.1 | 216.9 | 130.9 |
| 95 | Other | 0.2 | 0.2 | 17 | 133.4 |
| 96 | Other | 0.36 | 1.16 | 2.5 | 45.83 |
| 97 | Other | 0.1 | 0.1 | 13 | 108.6 |
| 98 | Other | 0.36 | 2.21 | 9 | 19 |
| 99 | Other | 0.1 | 0.1 | 10.3 | 129.1 |
| 100 | Other | 0.1 | - | 16.99 | 24.16 |
| 101 | Other | 0.07 | 0.3 | 7.0 | 131.9 |
